# Febrile Infection-Related Epilepsy Syndrome (FIRES) in a Young Adult: A Case Report Highlighting Advanced Neuroimaging

**DOI:** 10.1007/s00062-025-01574-9

**Published:** 2025-10-07

**Authors:** Evamaria Olga Riedel, Fabian Bongratz, Paul Theo Zebhauser, Mark Mühlau, Christian Wachinger, Dennis Martin Hedderich

**Affiliations:** 1 Department of Diagnostic and Interventional Neuroradiology, School of Medicine and Health, TUM Klinikum Rechts der Isar, Technical University of Munich, Munich, Germany; 2 Department of Radiology, School of Medicine and Health, Technical University of Munich, Munich, Germany; 3 Munich Center for Machine Learning, Munich, Germany; 4 Department of Neurology, School of Medicine and Health, TUM Klinikum Rechts der Isar, Technical University of Munich, Munich, Germany

## Introduction

Febrile infection-related epilepsy syndrome (FIRES) is a rare and severe subtype of new-onset refractory status epilepticus (NORSE), typically affecting previously healthy children and young adults. It usually follows a nonspecific febrile illness and progresses rapidly to refractory status epilepticus, often without an identifiable infectious agent. The pathogenesis remains unclear but likely involves an infection-triggered inflammatory response [1]. Diagnosis is often delayed due to overlap with other causes of new-onset seizures, including autoimmune encephalitis, infectious encephalitis, other infection-induced encephalopathies, and toxic-metabolic disturbances. MRI may aid in diagnosis, with bilateral T2-hyperintensities in the claustrum (“claustrum sign”) being a characteristic, though not pathognomonic, finding. While anti-seizure medications alone are often insufficient, immunomodulatory therapies including corticosteroids, IVIG, and cytokine-targeted therapies—as well as other supportive approaches—have shown promise and have contributed to the development of a consensus-based treatment framework [2]. The outcome is often poor and frequently associated with long-term cognitive impairment and chronic epilepsy [3].

This case adds to the growing body of literature on young adult-onset FIRES, and the potential benefit of early immunotherapy. Furthermore, advanced neuroimaging was useful in monitoring disease evolution and resolution in this patient.

## Case Presentation

A 23-year-old female with borderline personality disorder and a recent upper respiratory tract infection was admitted to the ICU following suspected suicidal ingestion of diphenhydramine and ibuprofen. She presented with generalized tonic-clonic seizures, minor impairment of consciousness (GCS 13), and subfebrile temperature (38.0 °C). No focal neurological signs were observed. Laboratory testing showed mild leukocytosis (9.3 G/L), elevated CRP (4.8 mg/dL; normal: < 0.5 mg/dL), increased CK (411 U/l), elevated creatinine (1.8 mg/dL) and reduced GFR, (39 mL/min). Toxicologic screening confirmed intoxication with diphenhydramine and high ibuprofen levels.

Initial treatment included midazolam, levetiracetam, and empirical antivirals/antibiotics (acyclovir, ceftriaxone, ampicillin). CSF analysis on day 6 revealed mild pleocytosis (48 cells/µL) with normal total protein, glucose, and lactate levels; microbiology and virology were negative. Repeat lumbar puncture on day 9 showed reduced cell count (10/µL) and continued normal total protein, glucose, and lactate levels as well as negative microbiology and virology. In the case of a not entirely excludable autoimmune encephalitis, no abnormalities were found in the corresponding comprehensive antibody diagnostics in serum and cerebrospinal fluid (*Supplement A1*). A borderline titer for RGS8, associated with paraneoplastic cerebellar syndrome in lymphoma, and found only in the blood, was considered nonspecific. Nonetheless we conducted CT imaging of the thorax, abdomen, and pelvis, along with a gynecological examination, which revealed no evidence of malignancy. Infectious workup—JC virus, Chlamydia pneumoniae and psittaci, Coxiella burnetii, Mycoplasma pneumoniae, Bartonella henselae, Shigella, HIV/hepatitis, syphilis, Borrelia, Leptospira, and Bornavirus—along with serological (serum electrophoresis, vasculitis, sarcoidosis) and metabolic evaluations (thyroid function, TRAK, Tg, TPO autoantibodies, cold agglutinins, vitamins B1 and B12, methylmalonic acid, holotranscobalamin, calcium, phosphate, magnesium) were unremarkable, except for mildly reduced free T3 levels.

Despite escalating antiseizure therapy with midazolam, levetiracetam, and later lacosamide, the patient had recurrent generalized and focal seizures with orofacial automatisms. Initial EEG demonstrated moderate to severe generalized background slowing without clear ictal activity. A repeat EEG later in the course revealed right-hemispheric polyspike activity consistent with focal epileptiform discharges, prompting the substitution of valproate for lacosamide.

On day 10, the patient developed fever (39 °C), tachycardia (125/min), leukocytosis (16.3 G/L), and a marked CRP elevation (17.1 mg/dL), without identifiable infectious focus. Previously de-escalated antibiotic therapy (ceftriaxone alone) was escalated to piperacillin/tazobactam.

**Fig. 1:**
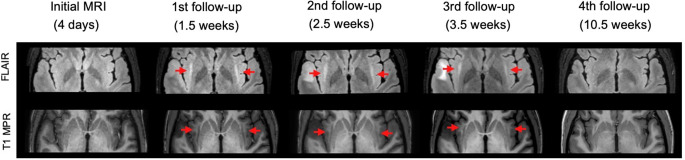
Evolution of the bilateral claustrum sign across serial MRI scans. The sign was absent on the initial MRI (4 days post-event), became apparent at the first follow-up MRI (1.5 weeks post-event), and persisted through the second and third follow-up MRI (2.5 and 3.5 weeks). It was no longer visible at the final follow-up MRI (10.5 weeks)

**Fig. 2:**
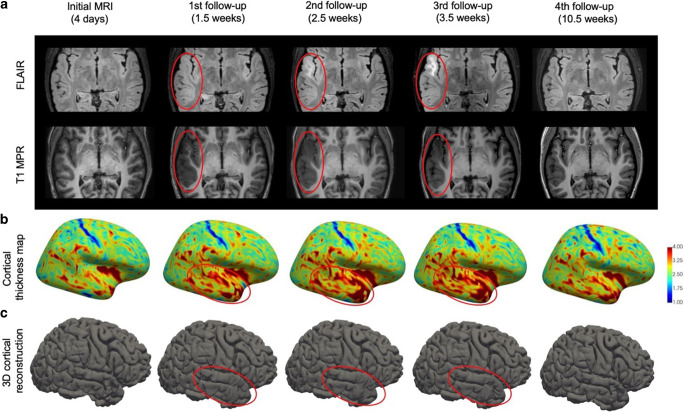
**a** Serial MRI scans obtained at baseline (4 days post-event) and at follow-up intervals at 1.5, 2.5, 3.5, and 10.5 weeks post-event. Cortical swelling in the right frontal and temporal operculum were observed at 1.5 weeks days post-event (first follow-up MRI), persisted through second and third follow-up at 2.5 and 3.5 weeks with concurrent subcortical edema and showed significant regression by 10.5 weeks. Progression and regression of cortical swelling can be seen in **b** cortical thickness maps and **c** 3D cortical surface reconstructions, which were generated by registering structural MRI data to the MNI152 space, cortical surface reconstruction in Vox2Cortex-Flow, manual revision for accuracy and visualization on an inflated FsAverage template using PyVista in Python

Initial noncontrast CT was unremarkable. First contrast-enhanced 3T MRI on day 4 retrospectively revealed subtle cortical thickening and blurring of the grey-white matter interface. MRI at 1.5 weeks revealed bilateral claustrum signs (Fig. 1), new focal cortical swelling in the right frontal and temporal operculum (Fig. 2) and swelling of the right hippocampus. Based on the bilateral claustrum sign, a diagnosis of FIRES was suspected.

High-dose IV methylprednisolone (1 g/day for 5 days) was initiated. The patient improved rapidly, regaining alertness and communication within two days.

Follow-up MRI at 2.5 and 3.5 weeks showed persistent claustrum signal, stable swelling in the right hippocampus and cortical right opercular regions and adjacent new but stable FLAIR edema.

The patient remained seizure-free after steroid tapering and was discharged on levetiracetam and lacosamide. She had no focal neurological deficits; MoCA score was 26/30 with mild attention and language deficits. At 10.5-week follow-up, she remained seizure-free under continued anti-seizure therapy, with normal EEG, laboratory and CSF findings. MRI showed resolution of claustrum signs and edema and significant regression of cortical swelling. At a five-month follow-up, no clear clinical signs of seizure recurrence were observed; no further neuropsychological testing was conducted.

## Discussion

Persistent seizures despite multiple antiseizure drugs, only transient response to benzodiazepines, and mild pleocytosis without infection or autoimmune markers are consistent with previously reported FIRES cases [4]. Initial MRI was unremarkable, as not uncommonly seen early in FIRES [5]. Subsequent imaging revealed typical features, including opercular and mesial temporal involvement and bilateral claustrum hyperintensities [6], which helped guide the diagnosis.

In this specific case, the advanced imaging tool Vox2Cortex-Flow [7]—a recent deep learning-based framework for accurate surface estimation—proved useful in visualizing the progression and regression of cortical swelling across follow-up MRIs.

Alternative etiologies were considered. The patient’s borderline personality disorder raised suspicion of psychogenic non-epileptic seizures, however EEG and MRI findings excluded this. Toxic effects from diphenhydramine and ibuprofen were evaluated, though these are not typically associated to seizures. The subfebrile temperature may have been postictal or due to diphenhydramine’s anticholinergic effects.

The patient’s favorable response to high-dose corticosteroids supports a possible autoimmune or inflammatory component—despite negative antibody testing—and antibody-negative autoimmune encephalitis or autoimmune epilepsy were considered. A beneficial effect of immunosuppressive therapy, particularly corticosteroids, has consistently been reported in FIRES [8] and is proposed as a first-line immunological intervention during the acute phase, as well as in post-acute management. Other immunomodulatory therapies such as IVIG as an alternative fist-line immunological treatment, and ketogenic diet initiated in the first week, are recommended during the acute phase. In the post-acute phase, ketogenic diet and if effective in the acute phase immunomodulation should be continued, vagus nerve stimulation may be beneficial [2].

The claustrum sign described above has been reported to be more characteristic of FIRES than autoimmune encephalitis [9]. Additionally, the absence of a neuropsychiatric prodrome—frequently present in autoimmune encephalitis—and the acute onset with numerous refractory seizures following a previous febrile infection favor FIRES as the more likely diagnosis.

Although long-term outcomes in FIRES are often poor, with epilepsy and cognitive impairments frequently reported [10], this patient remained seizure-free at 10.5 weeks. EEG and CSF findings were normal, and follow-up MRI showed regression of prior abnormalities. Interpretation of the outcome is complicated by episodes most consistent with dissociative seizures, likely related to her psychiatric comorbidity. At a five-month follow-up, no clear clinical signs of seizure recurrence were observed; but ongoing neuropsychological follow-up may be beneficial given the known risk of long-term impairments in FIRES.

The absence of confirmatory biomarkers limits diagnostic certainty, long-term outcomes remain unclear, and as a single case report generalizability is restricted; while advanced neuroimaging proved useful in this case, its broader applicability requires further validation.

## Conclusion

This case illustrates the diagnostic value of bilateral claustrum signs in FIRES in a young adult and highlights the importance of early recognition and a multidisciplinary approach when conventional anti-seizure therapies fail. In this specific case, advanced neuroimaging techniques such as cortical thickness mapping and 3D reconstruction proved useful for visualizing disease evolution and resolution. As FIRES remains a rare and challenging condition, further research is needed to improve understanding and guide treatment.

## Supplementary Information

A1: Antibody Diagnostics in Serum and Cerebrospinal Fluid
