# Supplementary material for: Febrile Infection-Related Epilepsy Syndrome (FIRES) in a Young Adult: A Case Report Highlighting Advanced Neuroimaging
Source: Clin Neuroradiol. 2025 Oct 7;36(2):771–4. doi: 10.1007/s00062-025-01574-9 (PMC13320097; doi:10.1007/s00062-025-01574-9)
Supplement: Supplementary file 1 — A1: Antibody Diagnostics in Serum and Cerebrospinal Fluid [file 62_2025_1574_MOESM1_ESM.pdf]

Supplement to:
Febrile Infection-Related Epilepsy Syndrome (FIRES) in a young adult: a case report highlighting advanced neuroimaging

A1: Antibody Diagnostics in Serum and Cerebrospinal Fluid

Cerebrospinal Fluid – IgG Antibodies Tested:
ANNA-3
Anti-glial nuclear antibodies (AGNA)
Amphiphysin
AP3B2
ARHGAP26
CASPR2
CDR2L
CV2 (CRMP-5)
DNER
GAD 65
Glutamate receptor (AMPA type 1/2)
Glutamate receptor (NMDA type)
Homer-3
Hu (ANNA-1)
IgLON5
LGI1
Ma (Ma1, Ma2/Ta)
PCA-2 (Purkinje cell cytoplasmic)
PNMA2
PRKCG
Recoverin
RGS8
Ri (ANNA-2)
RYR2
SOX1
Titin
Tr (Purkinje cell cytoplasmic)
Yo (PCA-1)
Zic4

Serum – IgG Antibodies Tested:
AP3B2
ARHGAP26
CDR2L
DNER
Glutamate receptor (AMPA type 1/2)
Homer-3
IgLON5
PRKCG
RGS8
RYR2
Yo
Zic4
